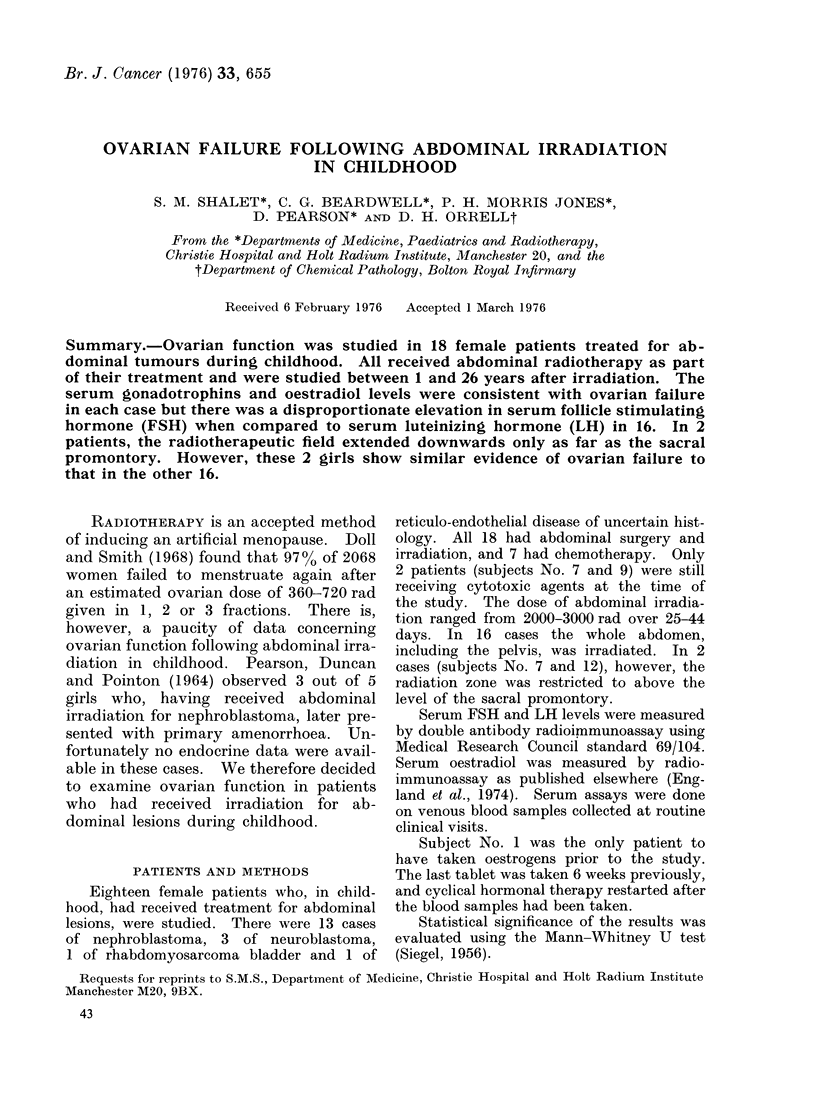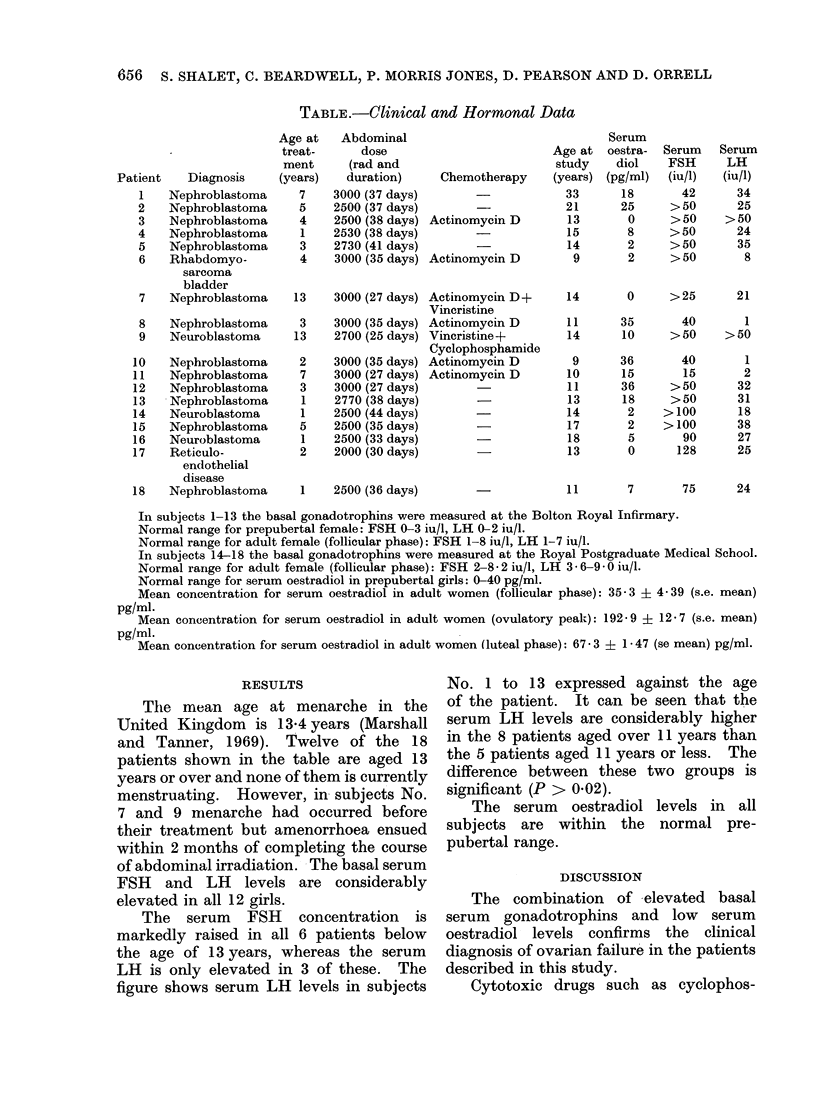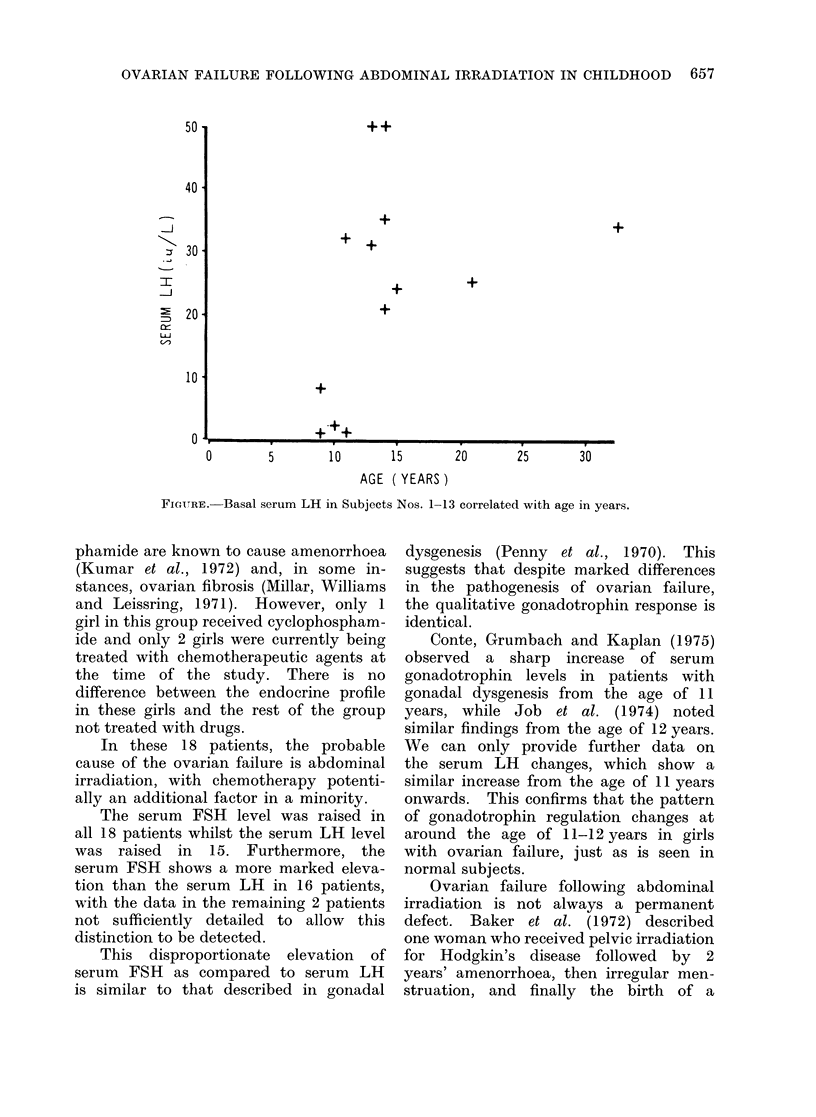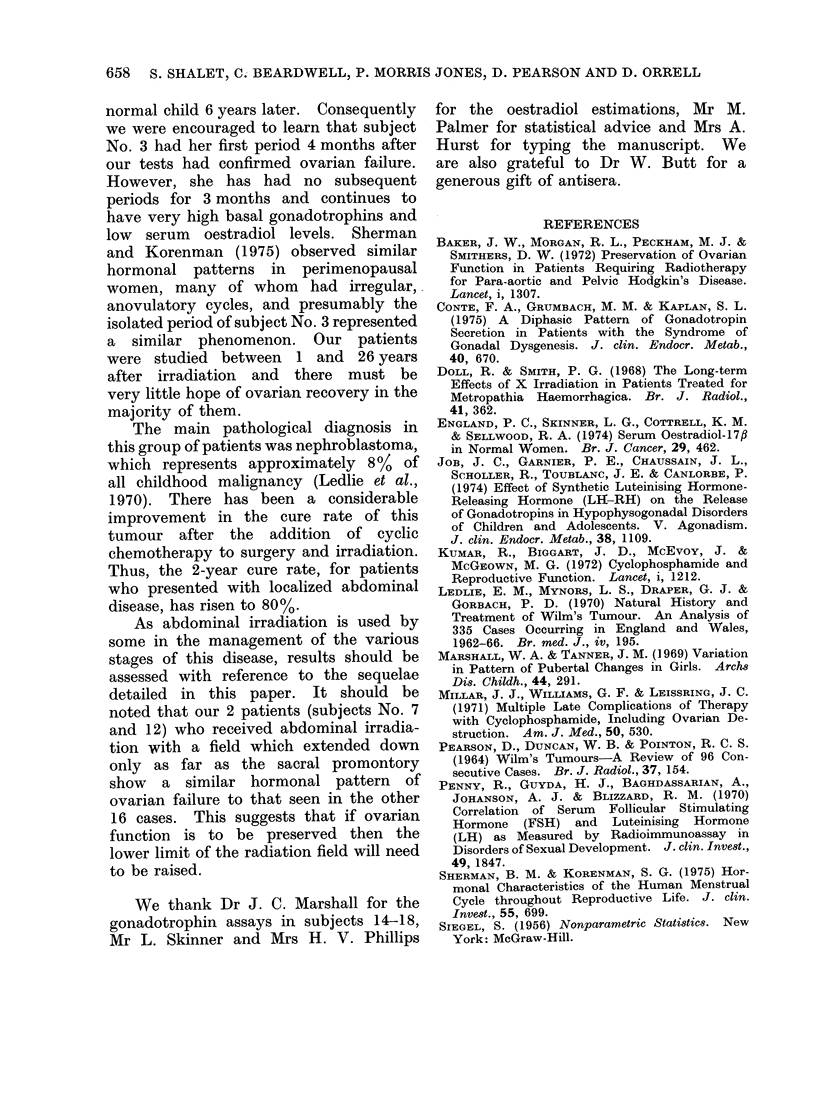# Ovarian failure following abdominal irradiation in childhood.

**DOI:** 10.1038/bjc.1976.103

**Published:** 1976-06

**Authors:** S. M. Shalet, C. G. Beardwell, P. H. Jones, D. Pearson, D. H. Orrell

## Abstract

Ovarian function was studied in 18 female patients treated for abdominal tumours during childhood. All received abdominal radiotherapy as part of their treatment and were studied between 1 and 26 years after irradiation. The serum gonadotrophins and oestradiol levels were consistent with ovarian failure in each case but there was a disproportionate elevation in serum follicle stimulating hormone (FSH) when compared to serum luteinizing hormone (LH) in 16. In 2 patients, the radiotherapeutic field extended downwards only as far as the sacral promontory. However, these 2 girls show similar evidence of ovarian failure to that in the other 16.


Br. J. Cancer (1976) 33, 655

### OVARIAN FAILURE FOLLOWING ABDOMINAL IRRADIATION IN CHILDHOOD

S. M. SHALET*, C. G. BEARDWELL*, P. H. MORRIS JONES*, D. PEARSON* AND D. H. ORRELL†

*From the *Departments of Medicine, Paediatrics and Radiotherapy, Christie Hospital and Holt Radium Institute, Manchester 20, and the †Department of Chemical Pathology, Bolton Royal Infirmary*

Received 6 February 1976 Accepted 1 March 1976

**Summary.—Ovarian function was studied in 18 female patients treated for abdominal tumours during childhood. All received abdominal radiotherapy as part of their treatment and were studied between 1 and 26 years after irradiation. The serum gonadotrophins and oestradiol levels were consistent with ovarian failure in each case but there was a disproportionate elevation in serum follicle stimulating hormone (FSH) when compared to serum luteinizing hormone (LH) in 16. In 2 patients, the radiotherapeutic field extended downwards only as far as the sacral promontory. However, these 2 girls show similar evidence of ovarian failure to that in the other 16.**

RADIOTHERAPY is an accepted method of inducing an artificial menopause. Doll and Smith (1968) found that 97% of 2068 women failed to menstruate again after an estimated ovarian dose of 360–720 rad given in 1, 2 or 3 fractions. There is, however, a paucity of data concerning ovarian function following abdominal irradiation in childhood. Pearson, Duncan and Pointon (1964) observed 3 out of 5 girls who, having received abdominal irradiation for nephroblastoma, later presented with primary amenorrhoea. Unfortunately no endocrine data were available in these cases. We therefore decided to examine ovarian function in patients who had received irradiation for abdominal lesions during childhood.

### PATIENTS AND METHODS

Eighteen female patients who, in childhood, had received treatment for abdominal lesions, were studied. There were 13 cases of nephroblastoma, 3 of neuroblastoma, 1 of rhabdomyosarcoma bladder and 1 of reticulo-endothelial disease of uncertain histology. All 18 had abdominal surgery and irradiation, and 7 had chemotherapy. Only 2 patients (subjects No. 7 and 9) were still receiving cytotoxic agents at the time of the study. The dose of abdominal irradiation ranged from 2000–3000 rad over 25–44 days. In 16 cases the whole abdomen, including the pelvis, was irradiated. In 2 cases (subjects No. 7 and 12), however, the radiation zone was restricted to above the level of the sacral promontory.

Serum FSH and LH levels were measured by double antibody radioimmunoassay using Medical Research Council standard 69/104. Serum oestradiol was measured by radioimmunoassay as published elsewhere (England *et al.*, 1974). Serum assays were done on venous blood samples collected at routine clinical visits.

Subject No. 1 was the only patient to have taken oestrogens prior to the study. The last tablet was taken 6 weeks previously, and cyclical hormonal therapy restarted after the blood samples had been taken.

Statistical significance of the results was evaluated using the Mann–Whitney U test (Siegel, 1956).

Requests for reprints to S.M.S., Department of Medicine, Christie Hospital and Holt Radium Institute Manchester M20, 9BX.

TABLE.—*Clinical and Hormonal Data*

| Patient | Diagnosis | Age at treatment (years) | Abdominal dose (rad and duration) | Chemotherapy | Age at study (years) | Serum oestradiol (pg/ml) | Serum FSH (iu/l) | Serum LH (iu/l) |
|---|---|---|---|---|---|---|---|---|
| 1 | Nephroblastoma | 7 | 3000 (37 days) | — | 33 | 18 | 42 | 34 |
| 2 | Nephroblastoma | 5 | 2500 (37 days) | — | 21 | 25 | >50 | 25 |
| 3 | Nephroblastoma | 4 | 2500 (38 days) | Actinomycin D | 13 | 0 | >50 | >50 |
| 4 | Nephroblastoma | 1 | 2530 (38 days) | — | 15 | 8 | >50 | 24 |
| 5 | Nephroblastoma | 3 | 2730 (41 days) | — | 14 | 2 | >50 | 35 |
| 6 | Rhabdomyosarcoma bladder | 4 | 3000 (35 days) | Actinomycin D | 9 | 2 | >50 | 8 |
| 7 | Nephroblastoma | 13 | 3000 (27 days) | Actinomycin D+ Vincristine | 14 | 0 | >25 | 21 |
| 8 | Nephroblastoma | 3 | 3000 (35 days) | Actinomycin D | 11 | 35 | 40 | 1 |
| 9 | Neuroblastoma | 13 | 2700 (25 days) | Vincristine+ Cyclophosphamide | 14 | 10 | >50 | >50 |
| 10 | Nephroblastoma | 2 | 3000 (35 days) | Actinomycin D | 9 | 36 | 40 | 1 |
| 11 | Nephroblastoma | 7 | 3000 (27 days) | Actinomycin D | 10 | 15 | 15 | 2 |
| 12 | Nephroblastoma | 3 | 3000 (27 days) | — | 11 | 36 | >50 | 32 |
| 13 | Nephroblastoma | 1 | 2770 (38 days) | — | 13 | 18 | >50 | 31 |
| 14 | Neuroblastoma | 1 | 2500 (44 days) | — | 14 | 2 | >100 | 18 |
| 15 | Nephroblastoma | 5 | 2500 (35 days) | — | 17 | 2 | >100 | 38 |
| 16 | Neuroblastoma | 1 | 2500 (33 days) | — | 18 | 5 | 90 | 27 |
| 17 | Reticulo-endothelial disease | 2 | 2000 (30 days) | — | 13 | 0 | 128 | 25 |
| 18 | Nephroblastoma | 1 | 2500 (36 days) | — | 11 | 7 | 75 | 24 |

In subjects 1–13 the basal gonadotrophins were measured at the Bolton Royal Infirmary.
Normal range for prepubertal female: FSH 0–3 iu/l, LH 0–2 iu/l.
Normal range for adult female (follicular phase): FSH 1–8 iu/l, LH 1–7 iu/l.
In subjects 14–18 the basal gonadotrophins were measured at the Royal Postgraduate Medical School.
Normal range for adult female (follicular phase): FSH 2–8·2 iu/l, LH 3·6–9·0 iu/l.
Normal range for serum oestradiol in prepubertal girls: 0–40 pg/ml.
Mean concentration for serum oestradiol in adult women (follicular phase): 35·3 ± 4·39 (s.e. mean) pg/ml.
Mean concentration for serum oestradiol in adult women (ovulatory peak): 192·9 ± 12·7 (s.e. mean) pg/ml.
Mean concentration for serum oestradiol in adult women (luteal phase): 67·3 ± 1·47 (se mean) pg/ml.

### RESULTS

The mean age at menarche in the United Kingdom is 13·4 years (Marshall and Tanner, 1969). Twelve of the 18 patients shown in the table are aged 13 years or over and none of them is currently menstruating. However, in subjects No. 7 and 9 menarche had occurred before their treatment but amenorrhoea ensued within 2 months of completing the course of abdominal irradiation. The basal serum FSH and LH levels are considerably elevated in all 12 girls.

The serum FSH concentration is markedly raised in all 6 patients below the age of 13 years, whereas the serum LH is only elevated in 3 of these. The figure shows serum LH levels in subjects No. 1 to 13 expressed against the age of the patient. It can be seen that the serum LH levels are considerably higher in the 8 patients aged over 11 years than the 5 patients aged 11 years or less. The difference between these two groups is significant ($P > 0{\cdot}02$).

The serum oestradiol levels in all subjects are within the normal prepubertal range.

### DISCUSSION

The combination of elevated basal serum gonadotrophins and low serum oestradiol levels confirms the clinical diagnosis of ovarian failure in the patients described in this study.

Cytotoxic drugs such as cyclophos-

FIGURE.—Basal serum LH in Subjects Nos. 1–13 correlated with age in years.

phamide are known to cause amenorrhoea (Kumar *et al.*, 1972) and, in some instances, ovarian fibrosis (Millar, Williams and Leissring, 1971). However, only 1 girl in this group received cyclophosphamide and only 2 girls were currently being treated with chemotherapeutic agents at the time of the study. There is no difference between the endocrine profile in these girls and the rest of the group not treated with drugs.

In these 18 patients, the probable cause of the ovarian failure is abdominal irradiation, with chemotherapy potentially an additional factor in a minority.

The serum FSH level was raised in all 18 patients whilst the serum LH level was raised in 15. Furthermore, the serum FSH shows a more marked elevation than the serum LH in 16 patients, with the data in the remaining 2 patients not sufficiently detailed to allow this distinction to be detected.

This disproportionate elevation of serum FSH as compared to serum LH is similar to that described in gonadal dysgenesis (Penny *et al.*, 1970). This suggests that despite marked differences in the pathogenesis of ovarian failure, the qualitative gonadotrophin response is identical.

Conte, Grumbach and Kaplan (1975) observed a sharp increase of serum gonadotrophin levels in patients with gonadal dysgenesis from the age of 11 years, while Job *et al.* (1974) noted similar findings from the age of 12 years. We can only provide further data on the serum LH changes, which show a similar increase from the age of 11 years onwards. This confirms that the pattern of gonadotrophin regulation changes at around the age of 11–12 years in girls with ovarian failure, just as is seen in normal subjects.

Ovarian failure following abdominal irradiation is not always a permanent defect. Baker *et al.* (1972) described one woman who received pelvic irradiation for Hodgkin's disease followed by 2 years' amenorrhoea, then irregular menstruation, and finally the birth of a normal child 6 years later. Consequently we were encouraged to learn that subject No. 3 had her first period 4 months after our tests had confirmed ovarian failure. However, she has had no subsequent periods for 3 months and continues to have very high basal gonadotrophins and low serum oestradiol levels. Sherman and Korenman (1975) observed similar hormonal patterns in perimenopausal women, many of whom had irregular, anovulatory cycles, and presumably the isolated period of subject No. 3 represented a similar phenomenon. Our patients were studied between 1 and 26 years after irradiation and there must be very little hope of ovarian recovery in the majority of them.

The main pathological diagnosis in this group of patients was nephroblastoma, which represents approximately 8% of all childhood malignancy (Ledlie *et al.*, 1970). There has been a considerable improvement in the cure rate of this tumour after the addition of cyclic chemotherapy to surgery and irradiation. Thus, the 2-year cure rate, for patients who presented with localized abdominal disease, has risen to 80%.

As abdominal irradiation is used by some in the management of the various stages of this disease, results should be assessed with reference to the sequelae detailed in this paper. It should be noted that our 2 patients (subjects No. 7 and 12) who received abdominal irradiation with a field which extended down only as far as the sacral promontory show a similar hormonal pattern of ovarian failure to that seen in the other 16 cases. This suggests that if ovarian function is to be preserved then the lower limit of the radiation field will need to be raised.

We thank Dr J. C. Marshall for the gonadotrophin assays in subjects 14–18, Mr L. Skinner and Mrs H. V. Phillips for the oestradiol estimations, Mr M. Palmer for statistical advice and Mrs A. Hurst for typing the manuscript. We are also grateful to Dr W. Butt for a generous gift of antisera.

### REFERENCES

BAKER, J. W., MORGAN, R. L., PECKHAM, M. J. & SMITHERS, D. W. (1972) Preservation of Ovarian Function in Patients Requiring Radiotherapy for Para-aortic and Pelvic Hodgkin's Disease. *Lancet*, i, 1307.

CONTE, F. A., GRUMBACH, M. M. & KAPLAN, S. L. (1975) A Diphasic Pattern of Gonadotropin Secretion in Patients with the Syndrome of Gonadal Dysgenesis. *J. clin. Endocr. Metab.*, **40**, 670.

DOLL, R. & SMITH, P. G. (1968) The Long-term Effects of X Irradiation in Patients Treated for Metropathia Haemorrhagica. *Br. J. Radiol.*, **41**, 362.

ENGLAND, P. C., SKINNER, L. G., COTTRELL, K. M. & SELLWOOD, R. A. (1974) Serum Oestradiol-17$\beta$ in Normal Women. *Br. J. Cancer*, **29**, 462.

JOB, J. C., GARNIER, P. E., CHAUSSAIN, J. L., SCHOLLER, R., TOUBLANC, J. E. & CANLORBE, P. (1974) Effect of Synthetic Luteinising Hormone-Releasing Hormone (LH–RH) on the Release of Gonadotropins in Hypophysogonadal Disorders of Children and Adolescents. V. Agonadism. *J. clin. Endocr. Metab.*, **38**, 1109.

KUMAR, R., BIGGART, J. D., MCEVOY, J. & MCGEOWN, M. G. (1972) Cyclophosphamide and Reproductive Function. *Lancet*, i, 1212.

LEDLIE, E. M., MYNORS, L. S., DRAPER, G. J. & GORBACH, P. D. (1970) Natural History and Treatment of Wilm's Tumour. An Analysis of 335 Cases Occurring in England and Wales, 1962–66. *Br. med. J.*, *iv*, 195.

MARSHALL, W. A. & TANNER, J. M. (1969) Variation in Pattern of Pubertal Changes in Girls. *Archs Dis. Childh.*, **44**, 291.

MILLAR, J. J., WILLIAMS, G. F. & LEISSRING, J. C. (1971) Multiple Late Complications of Therapy with Cyclophosphamide, Including Ovarian Destruction. *Am. J. Med.*, **50**, 530.

PEARSON, D., DUNCAN, W. B. & POINTON, R. C. S. (1964) Wilm's Tumours—A Review of 96 Consecutive Cases. *Br. J. Radiol.*, **37**, 154.

PENNY, R., GUYDA, H. J., BAGHDASSARIAN, A., JOHANSON, A. J. & BLIZZARD, R. M. (1970) Correlation of Serum Follicular Stimulating Hormone (FSH) and Luteinising Hormone (LH) as Measured by Radioimmunoassay in Disorders of Sexual Development. *J. clin. Invest.*, **49**, 1847.

SHERMAN, B. M. & KORENMAN, S. G. (1975) Hormonal Characteristics of the Human Menstrual Cycle throughout Reproductive Life. *J. clin. Invest.*, **55**, 699.

SIEGEL, S. (1956) *Nonparametric Statistics.* New York: McGraw-Hill.